# Assessment of serum diagnostic biomarkers for periprosthetic joint infection in hip and knee arthroplasty: a retrospective study

**DOI:** 10.7717/peerj.20294

**Published:** 2025-11-13

**Authors:** Zhiqiang Sun, Qiqi Zhang, Hui Ma, Xiaohe Wang, Changcheng Hua, Fei Yang

**Affiliations:** 1 Department of Orthopedics, The Affiliated Bozhou Hospital of Anhui Medical University, Bozhou City, Anhui Province, China; 2 Department of Orthopedics, The Affiliated Hospital of Jining Medical University, Jining City, Shandong Province, China

**Keywords:** Diagnosis, Revision surgery, Prosthesis-related infections, Platelet count, Mean platelet volume

## Abstract

**Background:**

Periprosthetic joint infection (PJI) after total hip and knee arthroplasty is challenging to differentiate from similar afflictions. Platelet count-to-mean platelet volume (PC/MPV) ratio has been proposed, but requires validation. This study evaluated PC/MPV and other potential serological biomarkers for diagnosing PJI prior to reimplantation arthroplasty of the hip and knee.

**Material and Methods:**

Medical records were retrospectively reviewed of patients who received hip and knee revision, and there were 88 PJI patients and 156 non-PJI patients met the modified 2018 criteria for inclusion. Receiver operating characteristic curves (ROCs) were used to analyze and compare the diagnostic performances of PC/MPV, fibrinogen (FIB), C-reactive protein (CRP), platelet count (PLT), erythrocyte sedimentation rate (ESR), and serum white blood cell (WBC) count.

**Results:**

Compared with the control group, the patients with PJI had significantly higher PC/MPV, FIB, ESR, CRP, serum WBC, and PLT, respectively, and the areas under the ROC curve were 0.787, 0.917, 0.832, 0.934, 0.685, and 0.778; that of FIB and CRP were similar. Regarding PC/MPV, the optimal cutoff was 27.81, and the sensitivity, specificity, and positive and negative predictive values were 0.807, 0.673, 0.582, and 0.861.

**Conclusions:**

The best diagnostic performance was achieved by CRP and FIB, and we recommend that these tests should be prioritized. Serum WBC, PC/MPV and PLT were insufficient to predict PJI prior to reimplantation arthroplasty of the hip and knee; however, CRP combined with FIB or PC/MPV best serves to obtain the most accurate prediction of PJI in our study.

## Background

Periprosthetic joint infection (PJI) is a serious complication after total hip and knee arthroplasty that can lead to pain, further surgeries, and huge economic burden (Kapadia et al., 2016). The rate of revision total knee and hip arthroplasty (from 3% to 8%) is expected to increase considerably from 1981 to 2014 (Schwartz et al., 2020; Seebach & Kubatzky, 2019). From 2000 to 2019, more people got hip and knee replacements, and that is expected to continue rising after 2020. With this trend and the growing costs of infection, revisions of these surgeries are likely to go up from 2014 to 2030 (Premkumar et al., 2021; Schwartz et al., 2020; Shichman et al., 2023). The PJI treatment methods include one-stage revision joint replacement surgery, two-stage revision joint replacement surgery, debridement with antibiotic and implant retention (DAIR), joint replacement removal and observation. Culturing negative results are common in clinical practice. According to Masaharu Watanabe’s research, around 25% of patients had negative cultures, often due to infections caused by less virulent organisms. These patients tend to have lower inflammatory markers, making it harder to diagnose infection before surgery. If cultures are negative, no resistant bacteria or soft tissue issues are present, a one-stage revision is done. For chronic infections, a two-stage process is standard: first, removing infected tissue and the prosthesis, then, after 6–12 weeks of antibiotics and no signs of infection, doing the revision surgery. Early infections are often managed with DAIR—removing infected tissue while keeping the implant and repairing soft tissues. If the patient’s condition is poor, removal or observation may be best (Watanabe et al., 2021). However, the diagnosis of PJI remains challenging because of the similarity between chronic low-grade infection and aseptic failure, in the context of chronic infection, patients often endure prolonged joint pain and restricted mobility, which can prove challenging to distinguish from aseptic failure (Gazendam et al., 2022).

Inflammatory markers are elevated in infection, rheumatoid arthritis and after surgery; on the other hand, the abnormal expressed of fibrinogen and other plasma proteins can increasing the measured markers (such as CRP, ESR and IL-6) (Saleh et al., 2018). We adopted the 2018 International Consensus Meeting (ICM 2018) as the diagnostic standard (Sousa et al., 2023). The presence of the same microorganisms in two cultures or the existence of sinus tracts was used as the main diagnostic criteria. Secondary criteria included elevated levels of serum CRP, D-dimer, ESR, synovial white blood cells, leukocyte esterase, alpha-defensin, and synovial CRP to evaluate the preoperative and postoperative comprehensive scores, thereby diagnosing PJI (Sousa et al., 2023). Although guidelines have improved, there is no gold-standard diagnostic test for PJI (Carli et al., 2019). Various biomarkers or technologies have been proposed, but finally no test is sufficiently specific or sensitive for PJI, including next generation sequencing, synovial calprotectin, synovial fluid IL-4, leukocyte esterase, serum D-lactate, or alpha-defensin (Chisari & Parvizi, 2020; Hantouly et al., 2022; Li et al., 2021; Miyamae et al., 2019). Although the precise diagnosis of PJI still faces many challenges, serum biomarkers have been proven to be a non-invasive, rapid and inexpensive method for preoperative auxiliary diagnosis. CRP, as a serum parameter for diagnosing PJI, has been supported by the European Bone and Joint Infection Society (EBJIS), the Musculoskeletal Infection Society (MSIS), and other societies (Moldovan, 2024). Tarabichi et al. (2024) compared four parameters, namely D-Dimer, ESR, CRP, and FIB, and concluded that all these parameters demonstrated comparable accuracy in diagnosing PJI. When the sensitivity was maximized to 100%, D-Dimer showed the highest specificity and was superior to the other three parameters for screening PJI. However, a single detection indicator alone cannot prove the diagnosis of PJI (Le et al., 2025). Diagnosis of PJI relies primarily on serological test (such as ESR,CRP, WBC, IL-6, PCT, D-Dimer, et al.), for its simplicity and accessibility. Hemogram indexes are the most common inflammatory markers because they are easy, cheap, and fast. Platelet count (PC) and mean platelet volume (MPV) are considered novel prognosis biomarkers in patients with many diseases, such as COVID-19, rheumatoid arthritis, Henoch-Schonlein purpura and viral respiratory infectious (Feketea et al., 2022; Liu et al., 2022; Pereckova et al., 2022; Xu et al., 2022a). However, there are some disadvantages in using only PC and MPV for diagnosing infection (Mishra et al., 2021). Sigmund et al. (2021) determined that platelet count-to-mean platelet volume (PC/MPV) may help differentiate PJI from asepsis, with sensitivity and specificity values of 0.427 and 0.812, respectively. However, the value of PC/MPV is debatable and requires validation (Klemt et al., 2022; Munoz-Mahamud et al., 2022). The present retrospective study explored the diagnostic value and best PC/MPV threshold for diagnosing PJI prior to reimplantation arthroplasty of the hip and knee, and evaluated other potential serological biomarkers.

## Materials and Methods

### Patients

**Figure 1:**
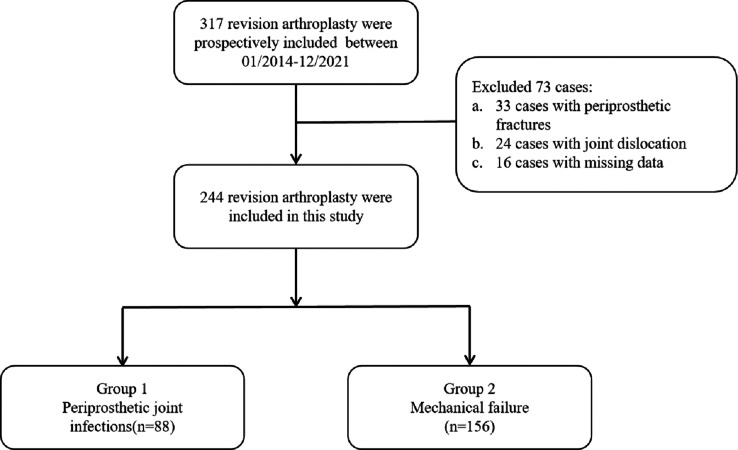
Inclusion and exclusion criteria of patients in the study design.

The records of 244 patients who underwent revision arthroplasty at our hospital from 2014 to 2021 were retrospectively reviewed (Fig. 1). The diagnosis of PJI was based on the validated evidence-based criteria for diagnosing PJI after hip and knee arthroplasty, published in 2018 (Table 1) (Parvizi et al., 2018). Patients were included if they had undergone revision surgery and had complete pre-operative laboratory and intra-operative synovial fluid culture results available. Exclusion criteria included periprosthetic fracture, joint dislocation of the arthroplasty, systemic lupus erythematosus, rheumatoid arthritis, systemic infection, malignant tumors, or heavy smoking (smoking more than 20 cigarettes per day for at least 5 years continuously) (Khudhur et al., 2025). The study was approved by the Ethics Committee of Affiliated Hospital of Jining Medical University (2022c228) and was conducted in accordance with the Declaration of Helsinki. The Institutional Review Board waived the requirement for written informed consent since this was a retrospective study.

**Table 1:** The evidence-based definition for periprosthetic joint infection in 2018.

	Diagnostic criteria	Score	Decision
Major criteria	1. Two positive cultures with the same organisms	–	Infected
	2. A sinus tract communicating with the joint	–	–
Minor criteria	1. Elevated serum CRP or D-Dimer	2	–
	2. Elevated serum ESR	1	≥6 Infected
	3. Elevated synovial WBC or LE	3	2–5 Possibly infected
	4. Positive alpha-defensin	3	0–1 Not infected
	5. Elevated synovial PMN (%)	2	–
	6. Elevated synovial CRP	1	–
Inconclusive*	1. Preoperative Score	–	≥6 Infected
	2. Positive histology	3	4–5 Inconclusive
	3. Positive purulence	3	≤3 Not infected
	4. Single positive culture	2	–

**Notes.**

* Inconclusive preop score or dry tap joint aspirate.

### Data collection

Comprehensive pre-operative data were systematically extracted from electronic medical records for all eligible patients. Data including gender, age, body mass index (BMI), the calculation of the platelet count to mean platelet volume ratio (PC/MPV), along with measurements of fibrinogen, erythrocyte sedimentation rate (ESR), C-reactive protein (CRP), white blood cell count (WBC), and platelet count (PLT). All laboratory analyses were performed using standardized automated analyzers in the hospital’s central laboratory. Specifically, plasma fibrinogen levels were quantified with an automated coagulation analyzer (Sysmex CS-2500; Sysmex Corporation, Japan) *via* the Clauss method. CRP was measured using particle-enhanced immunoturbidimetric assay on a Cobas c 501 analyzer (Roche Diagnostics, Basel, Switzerland) with a detection limit of 0.6 mg/L. ESR was determined using the Westergren method. Complete blood count parameters were analyzed on a Sysmex XN-9000 hematology analyzer (Sysmex Corporation, Kobe, Japan). The PC/MPV ratio was calculated as the absolute platelet count divided by MPV. Synovial fluid samples were collected aseptically during revision surgery and cultured in BACTECTM FX blood culture bottles using the BACTECTM 9120 system (Becton Dickinson, Franklin Lakes, NJ, USA).

Synovial fluid was aspirated aseptically during the revision surgery and was immediately sent for microbial culture and analysis. Standardized anteroposterior and lateral radiographs of the involved joint were also obtained and assessed pre-operatively for all patients.

### Statistical analysis

All statistical analyses were performed using IBM SPSS 23 (Armonk, NY, USA) or MedCalc 19.0.4 (Ostend, Belgium). Figures were produced using GraphPad Prism 8 (San Diego, CA, USA). Normality of continuous variables was assessed using the Shapiro–Wilk test. Quantitative data with normal distribution are presented as mean ± standard deviation and compared using independent samples *t*-tests. Non-normally distributed data are presented as median with interquartile range and compared using Mann–Whitney *U* test. Categorical variables are expressed as frequencies and compared *via* chi-square test. Receiver operating characteristic (ROC) curves and the area under the curve (AUC) were used to evaluate the diagnostic performance of each biomarker. Optimal cutoff values were determined using the Youden index. Comparison of ROC curves was performed using the DeLong test. A two-tailed *p*-value <0.05 was considered statistically significant.

## Results

**Figure 2:**
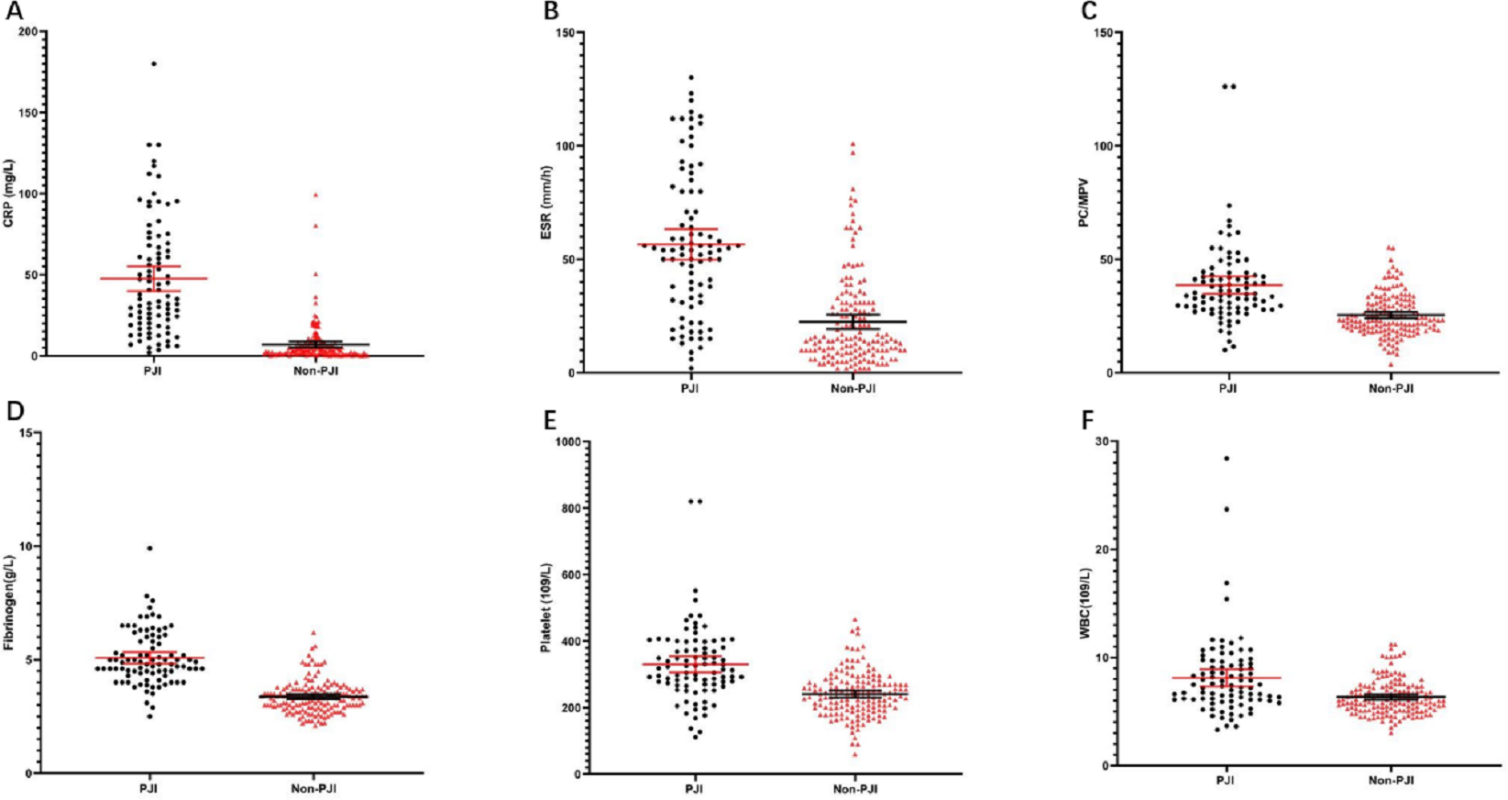
Distributions of PC/MPV, FIB, ESR, CRP, PLT count, and serum WBC count. The solid line represents the average and the 95% CI.

The study population comprised 244 patients who had received revision arthroplasty after total hip and knee replacement from January 2014 to December 2021 (Table 2). Among the 244 patients, 88 (36.1%) experienced PJI, leaving 156 in the non-PJI group. Between the PJI and non-PJI groups, there were no significant differences regarding sex, age, body mass index, surgical position, or American Society of Anesthesiologists (ASA) physical status classification. However, compared with the non-PJI group, the time to revision was significantly shorter in the PJI group (*P* < 0.001), and the patients with PJI had significantly higher levels of all the tested potential biomarkers (Table 3; Fig. 2), specifically: PC/MPV, FIB, ESR, and CRP (all, *P* < .001), and WBC and PLT (both, *P* < 0.05).

**Table 2:** Basic characteristics of patients in the study*.

		PJI	Non-PJI	***P*** ** value**
Subjects		88 (36.1)	156 (63.9)	
Gender	Male	41 (46.6%)	62 (39.7)	0.298
	Female	47 (53.4)	94 (61.3)	
Age, y		65.75 ± 9.54 (27–88)	64.55 ± 10.16 (26–86)	0.525
BMI, kg/m^2^		24.89 ± 4.50 (14.52–35.18)	25.65 ± 4.17 (16.63–37.11)	0.420
Affected joint	Knee	58 (66.0)	42 (28.0)	<0.001
	Hip	30 (34.0)	114 (72.0)	
Position	Left	42 (47.8)	83 (61.7)	0.410
	Right	46 (53.2)	73 (38.3)	
ASA	1	2 (2.3)	6 (3.8)	0.700
	2	47 (53.4)	78 (50.0)	
	3	35 (39.8)	68 (43.6)	
	4	4 (4.5)	4 (2.6)	
Time to revision, y		2.78 ± 3.91 (0.03–20)	7.99 ± 6.23 (0.05–30)	<0.001

**Notes.**

* Data are reported as *n* (%), unless indicated otherwise.

**Table 3:** The tested biomarkers in the PJI group and non-PJI group.

	PJI group	Non-PJI group	*P* ** value**
Fibrinogen, g/L	5.09 ± 1.19	3.40 ± 0.71	<0.001
ESR, mm/h	56.57 ± 31.86	22.65 ± 20.01	<0.001
CRP, mg/L	51.66 ± 45.53	7.00 ± 11.98	<0.001
PC/MPV	38.65 ± 18.1	25.43 ± 9.02	<0.001
WBC Count, 10^9^/L	8.11 ± 3.68	6.34 ± 1.60	<0.001
Platelet Count, 10^9^/L	330.50 ± 114.80	241.00 ± 66.88	<0.001

**Figure 3:**
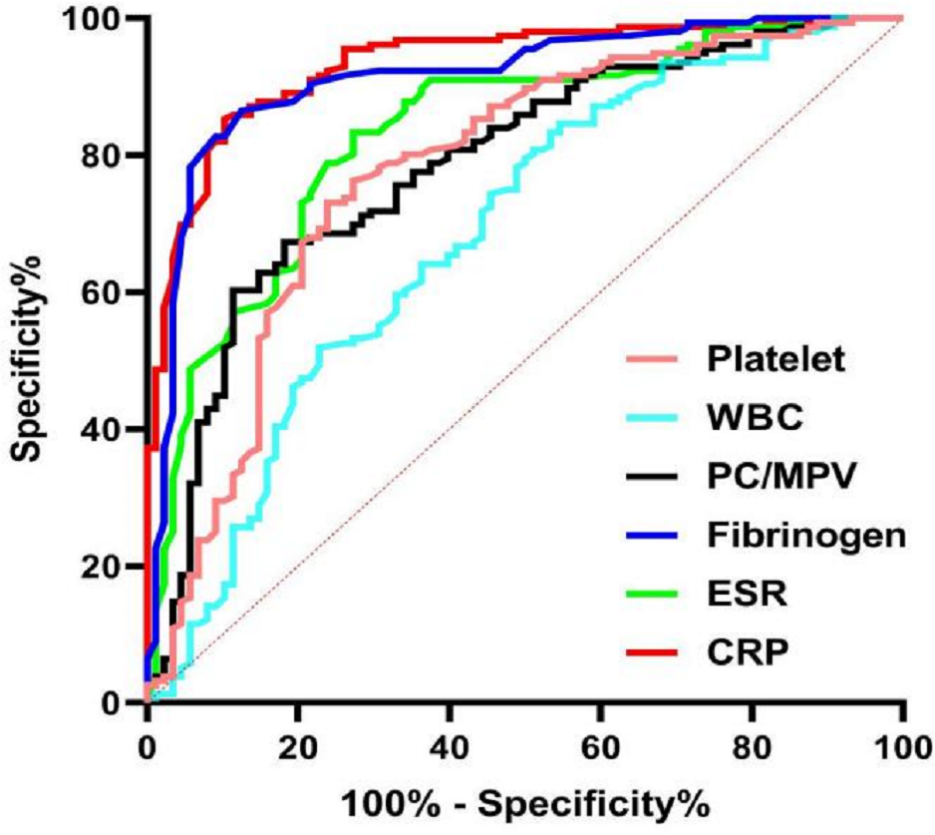
ROC curves of PC/MPV, FIB, ESR, CRP, PLT count, and serum WBC count for the preoperative diagnosis of PJI.

The ROC curves of PC/MPV, FIB, ESR, CRP, PLT, and serum WBC count for the diagnosis of PJI are shown in Fig. 3. For diagnosis of PJI, CRP had the highest AUC, and then FIB, ESR, PC/MPV, and PLT. The diagnostic performance of WBC was poor (Table 4). Moreover, see Table 4 for the comparative analysis of other biomarkers compared with CRP, the AUC values of CRP were comparable to that of FIB (*P* = 0.238).

**Table 4:** The diagnosis values and threshold of PC/MPV, plasma fibrinogen, ESR, CRP, Platelet count and serum WBC count for 244 cases.

Biomarks	AUC	95% CI	Youden index	Threshold	Sensitivity	Specificity	PPV	NPV	Compared with CRP
CRP	0.934	0.902–0.965	0.744	10.95 (mg/L)	0.898	0.853	0.775	0.937	/
Fibrinogen	0.914	0.876–0.953	0.760	3.95(g/L)	0.864	0.865	0.784	0.918	0.238
ESR	0.832	0.778–0.885	0.561	36.50 (mm/h)	0.739	0.833	0.714	0.850	<0.001
WBC	0.685	0.613–0.757	0.280	7.77(10^9^/L)	0.466	0.846	0.631	0.737	<0.001
PC/MPV	0.787	0.727–0.847	0.491	27.81	0.818	0.673	0.582	0.861	<0.001
Platelet	0.778	0.714–0.842	0.490	269.5(10^9^/L)	0.761	0.731	0.615	0.844	<0.001

**Figure 4:**
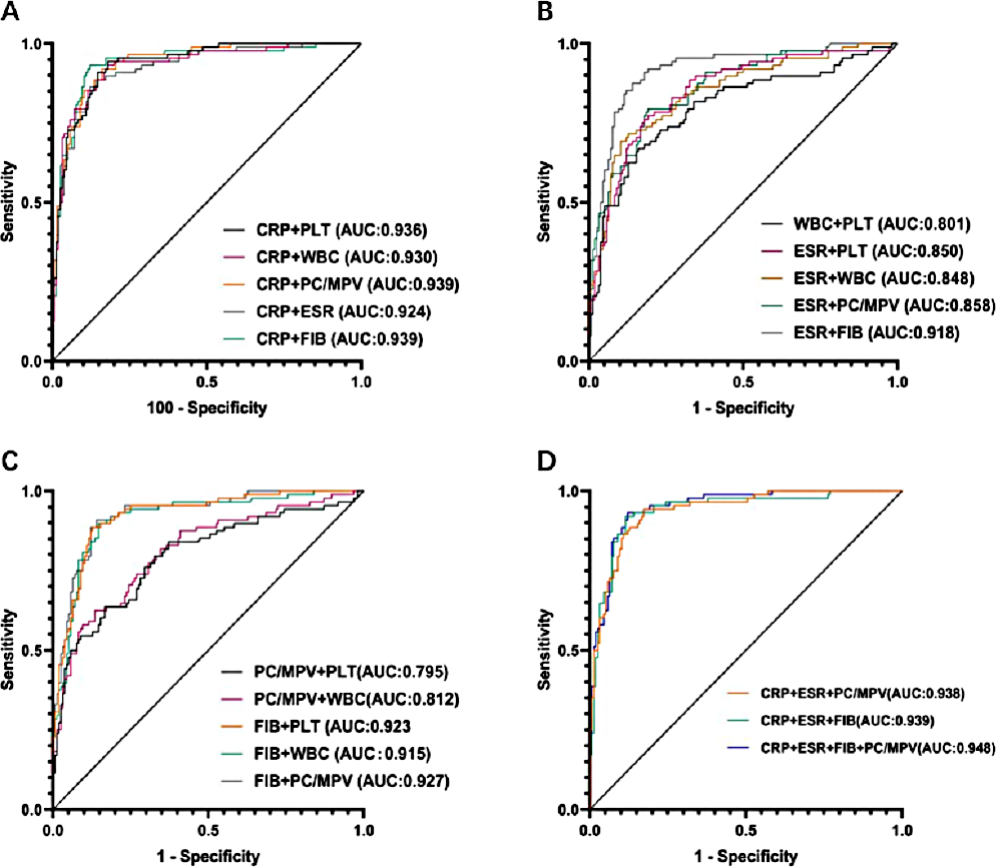
ROCs of paired biomarkers.

The calculated thresholds of PC/MPV, FIB, ESR, CRP, PLT and serum WBC count were 27.81, 3.95 g/L, 36.5 mm/h, 10.95 mg/L, 269.5 × 10^9^/L, 7.77 × 10^9^/L, respectively.The sensitivity, specificity, and positive and negative predictive values (PPV, NPV) are displayed in Table 4. The highest AUC values (0.939) were shown by CRP combined with FIB (sensitivity 0.932, specificity 0.878), and CRP combined with PC/MPV (sensitivity 0.92, specificity 0.84; Fig. 4).

When conducting subgroup analysis based on joint type and infection duration, all markers in the hip replacement patients showed significant increases in the PJI group (*P* < 0.01), among which CRP showed the largest difference (37.78 *vs* 8.67 mg/L, *P* < 0.001). The same differences were observed in the knee replacement patients (*P* < 0.01). The CRP of acute PJI patients was significantly higher than that of chronic PJI patients (66.10 *vs* 49.56 mg/L), but there was no inter-group difference in platelet count in the acute phase (*P* = 0.205). In chronic PJI patients, the average platelet volume (MPV) was significantly decreased (8.91 *vs* 9.82 fl, *P* < 0.001), and the PC/MPV index had no diagnostic value in the acute phase (*P* = 0.557) (Tables 5; 6).

**Table 5:** Subgroup analyses based on joint type.

Biomarks	Hip group (*n* = 148)			Knee group (*n* = 96)		
	PJI group (*n* = 32)	Non-PJI group (*n* = 116)	*P*	PJI group (*n* = 56)	Non-PJI group (*n* = 40)	*P*
WBC Count, 10^9^/L	7.37 ± 2.18	6.35 ± 1.66	0.002	8.42 ± 4.71	6.22 ± 1.92	<0.001
Platelet Count, 10^9^/L	318.84 ± 98.05	231.68 ± 65.32	<0.001	331.84 ± 143.27	258.55 ± 74.82	0.001
CRP, mg/L	37.78 ± 39.92	8.67 ± 15.32	<0.001	57.03 ± 62.19	7.65 ± 14.85	<0.001
ESR, mm/h	53.26 ± 31.08	22.46 ± 20.45	<0.001	61.30 ± 32.54	25.35 ± 22.67	<0.001
Fibrinogen, g/L	4.75 ± 1.10	3.43 ± 0.79	<0.001	5.04 ± 1.48	3.41 ± 0.79	<0.001
PC/MPV	35.26 ± 13.68	23.18 ± 9.07	<0.001	39.13 ± 24.83	27.92 ± 9.75	0.004

**Table 6:** Subgroup analyses based on infection chronicity.

Biomarks	Acute group (*n* = 32)			Chronic group (*n* = 212)		
	**PJI** group (*n* = 20)	**Non-PJI** group (*n* = 12)	***P***	**PJI** group (*n* = 68)	**Non-PJI** group (*n* = 144)	***P***
WBC Count, 10^9^/L	8.02 ± 2.93	5.82 ± 1.52	0.012	8.04 ± 4.40	6.29 ± 1.76	<0.001
Platelet Count, 10^9^/L	312.65 ± 103.58	273.42 ± 59.18	0.205	8.91 ± 1.16	9.82 ± 1.50	<0.001
CRP, mg/L	66.10 ± 78.32	6.98 ± 10.10	0.002	49.56 ± 53.19	7.25 ± 14.92	<0.001
ESR, mm/h	47.00 ± 25.49	28.18 ± 22.54	0.031	57.35 ± 31.78	22.58 ± 21.05	<0.001
Fibrinogen, g/L	4.99 ± 1.65	3.57 ± 0.82	0.004	4.95 ± 1.21	3.38 ± 0.77	<0.001
PC/MPV	35.97 ± 13.24	33.23 ± 11.94	0.557	38.40 ± 20.12	25.01 ± 9.41	<0.001

## Discussion

Periprosthetic joint infection (PJI) after total knee and hip arthroplasty is a rare but severe complication, and if not diagnosed early, can lead to catastrophic consequences (Kamath et al., 2015). Diagnostic biomarkers and technologies have been developed and have shown high sensitivity and specificity (Cipriano et al., 2012; Henkelmann et al., 2022; Hong et al., 2022). However, most of these cannot differentiate low-virulence infection, and rapid and accurate diagnosis remains challenging. Furthermore, the reported predictive cutoffs are inconsistent (Lum et al., 2018; Natsuhara et al., 2019). There is no single test or composite of variables that can rule out PJI. Comorbidities and clinical characteristics can affect the diagnostic value of tests and biomarkers. These include systemic lupus erythematosus, rheumatoid arthritis, systemic infection, malignant tumors, and heavy smoking. In the present study, potential subjects with any of these potential confounders were excluded (Huang et al., 2021).

Orthopedic surgeons generally prefer to diagnosis PJI by non-invasive testing. Preoperative serum tests are the most common because they are easy, cheap, and fast. CRP and ESR have been recommended in various guidelines as diagnostic indicators, with reportedly high sensitivity and specificity (Parvizi et al., 2018; Workgroup Convened by the Musculoskeletal Infection, 2011), with CRP sensitivity (specificity) 62 to 100% (64–96%), and ESR sensitivity (specificity) 33 to 95% (60–100%) (Sigmund, Puchner & Windhager, 2021). In the present study, the CRP and ESR provided acceptable diagnostic performance, with an AUC of 0.934 for CRP (0.898 sensitivity, 0.853 specificity) at a cutoff of 10.95 mg/L, and for ESR an AUC of 0.832 (0.739 sensitivity, 0.833 specificity) at a cutoff of 36.5 mm/h.The references provided by the Musculoskeletal Infection Society (MSIS), the Infectious Diseases Society of America (IDSA), and the International Consensus Meeting (ICM) for the diagnosis of PJI are CRP > 10 mg/L; ESR > 30 mm/h (Sousa et al., 2023). In our study, the diagnostic thresholds for CRP and ESR were slightly higher than this value. Yet, these systemic inflammatory proteins can be elevated in patients with comorbidities and the thresholds differ between hips and knees, and acute and chronic infection (Tirumala et al., 2021). In the subgroup analysis, CRP levels were 7.5 times higher in knee joint PJI and 4.4 times higher in hip joint PJI. The imbalance in the sample size of the subgroups (for instance, only 20 cases in acute PJI) may affect the statistical power. Future studies need to expand the cohort for verification.

Serum WBC count is a common measure used when diagnosing various diseases. Some studies found that the WBC count may be helpful to aid diagnosis of PJI, with the sensitivity of WBC ranging from 21% to 91%, and the specificity from 60% to 95%, it shows different sensitivities and specificities depending on the cutoff levels (Goud et al., 2022). In this study, the WBC count provided poor diagnostic performance, with an AUC of 0.658 (0.466 sensitivity, 0.846 specificity) at a cutoff value of 7.77 × 10^9^/L. The WBC count of the PJI group was higher than that of the non-PJI, and but its diagnostic value for PJI was lower than that of the other potential biomarkers PC/MPV, FIB, ESR, CRP, and PLT. Given these limitations, serum WBC count was not been included in any diagnostic guidelines. However, synovial WBC count is included in many guidelines for diagnosis of PJI, due to high sensitivity (Shahi et al., 2017).

FIB is a large (340 kDa) hexametric homodimer secreted by the liver. There are studies that suggest that FIB can be important in the diagnosis of PJI (Li et al., 2019b). In our previous study, we found that FIB showed promising diagnostic strength, with an AUC of 0.916 (0.860 sensitivity, 0.900 specificity) (Yang et al., 2021), comparable to that of CRP or ESR. Also, Xu et al. (2019) demonstrated that FIB was significantly higher in patients with PJI compared with patients without PJI. Xu et al. (2022b) found that FIB was a significant biomarker and reported a sensitivity of 0.696 and higher specificity of 0.865; the AUC of FIB was only inferior to that of CRP among serum biomarkers. The present results indicate that FIB is a promising biomarker for identifying PJI: at a calculated cutoff of 3.95 g/L, the AUC, sensitivity, specificity, PPV, and NPV were 0.914, 0.864, 0.865, 0.784, and 0.915, respectively. The diagnostic value of FIB was not significantly different (*P* = 0.238) from that of CRP, according to the DeLong test comparing the respective AUCs. The sensitivity and NPV of FIB and CRP were quite comparable, and could be utilized as screening tools.

PLTs are small anucleate cell fragments in the blood that can aid blood clotting through hemostasis and thrombosis (Deppermann & Kubes, 2016). Previously published studies showed that PLT levels differed among various infections or inflammatory diseases. For example, in a study that evaluated platelet indices for diagnosing deep surgical site infection (DSSI) after fixation for limb fractures, Zhang et al. (2018) found that PLT count and distribution width were significantly higher in the case group (with DSSI) relative to a control group (without DSSI), while the MPVs were similar. The authors concluded that platelet distribution width combined with PLT could be diagnostic. However, there are some disadvantages in using only PLT and MPV for diagnosing infection (Sahin et al., 2021). Strony et al. (2020) further assessed the ability of PC/PMV to diagnose fracture-related infection, and reported that the accuracies of PC/MPV, CRP, and ESR were similar. They recommended that PC/MPV may be a reliable screening test for infection. PC/MPV has been reported as biomarkers of head and neck cancer, oral cavity and septic shock (Liberski, Szewczyk & Krzych, 2020; Tham et al., 2019). Recent evidence suggested that PC/MPV may be a potential screening test before revision arthroplasty to predict PJI. According to Paziuk et al. (2020), the value of PC/MPV for predicting PJI was high (sensitivity 0.4810, specificity 0.8085, AUC 0.69), and while the diagnostic strength was less than that of either ESR or CRP, the specificity of PC/MPV was greater than either. Chen et al. (2022) reported that PC/MPV and PLT had limited value for diagnosing PJI in patients who had undergone total joint arthroplasty: for PC/MPV (PLT) the AUC, sensitivity, and specificity were 0.704 (0.703), 0.760 (0.657), and 0.593 (0.679), respectively.

However, in the present study, the AUCs of PC/MPV and PLT were 0.787 and 0.778. This suggests that PC/MPV and MPV had poor diagnostic accuracy for diagnosing PJI. The diagnostic accuracy greatly improved when PC/MPV was combined with CRP (AUC 0.939, sensitivity 0.92, specificity 0.84). The enhanced ability to diagnose PJI more accurately when combining CRP with either fibrinogen or the PC/MPV ratio stems from their complementary roles in the underlying disease process. CRP acts as a sensitive marker that indicates the overall inflammatory response in the body, mainly driven by IL-6 which prompts the liver to produce acute-phase proteins. Fibrinogen, which also rises during inflammation, directly contributes to the local clotting abnormalities seen in PJI, where bacteria forming biofilms lead to a hypercoagulable environment and fibrin buildup within the joint (Li et al., 2019a; Xu et al., 2021). By combining CRP and fibrinogen levels, clinicians can simultaneously assess the widespread inflammation and the local clotting activity associated with infection. Meanwhile, the value of the PC/MPV ratio, despite its moderate standalone performance, lies in reflecting how the body’s bone marrow responds to ongoing infection. When chronic inflammation takes hold, the body often responds with an increased platelet count, a process driven by pro-inflammatory cytokines. The fluctuations in mean platelet volume also mirror how platelet production and usage change over time (Klemt et al., 2022; Paziuk et al., 2020). Therefore, when you see high CRP levels indicating major inflammation, along with an altered PC/MPV ratio that points to a hematopoietic response, it offers a more specific confirmation of an active infection. This dual approach helps reduce false positives that could happen with non-infectious inflammation. In conclusion, PC/MPV, FIB, ESR, CRP, PLT and serum WBC count are useful predictor in the diagnosis of PJI due to the cost-effective and easy-to-assess nature in clinical practice, it can increase diagnostic accuracy.

### Limitations

This study is potentially limited by its single-center retrospective design, which could lead to biases. Secondly, and importantly, we lacked detailed data on preoperative antibiotic and anticoagulant therapy. The administration of antibiotics could suppress inflammatory responses, potentially leading to an underestimation of biomarker levels in some infected patients. Conversely, anticoagulants may influence platelet indices. The absence of this information is a source of potential confounding that must be considered when interpreting our results. Finally, the sample size remains a constraint common in PJI research due to the condition’s relative rarity. A *post-hoc* power analysis was performed for our primary biomarker, CRP. Given the analysis revealed a high statistical power, well above the conventional 80% threshold. This indicates that our study was adequately powered to detect the significant differences we reported for the main comparisons. However, the sample size limited our ability to perform robust subgroup analyses stratified by time of onset or joint location without risking type II errors. Indeed, our preliminary subgroup analysis suggested intriguing trends. Future multi-center studies with larger cohorts are essential to validate these exploratory subgroup findings and establish time-specific and joint-specific diagnostic thresholds.

## Conclusion

PC/MPV and PLT were insufficient as biomarkers to predict PJI prior to reimplantation arthroplasty of the hip and knee. The best diagnostic performance was achieved by CRP and FIB, and we recommend that these tests should be prioritized. CRP combined with FIB or PC/MPV would best serve to obtain the most accurate prediction of PJI.

##  Supplemental Information

10.7717/peerj.20294/supp-1 Supplemental Information 1 Raw data

10.7717/peerj.20294/supp-2 Supplemental Information 2 STROBE checklist

## Additional Information and Declarations

### Data Availability

The following information was supplied regarding data availability: The raw data are available in the Supplementary File.
