# Supplementary material for: Assessment of serum diagnostic biomarkers for periprosthetic joint infection in hip and knee arthroplasty: a retrospective study
Source: PeerJ. 2025 Nov 13;13:e20294. doi: 10.7717/peerj.20294 (PMC12619940; doi:10.7717/peerj.20294)
Supplement: Supplemental Information 2 [file peerj-13-20294-s002.doc]

STROBE Statement—Checklist of items that should be included in reports of ***case-control studies***

|  | Item No | Recommendation |
| --- | --- | --- |
| **Title and abstract** | 1 | (*a*) Page 1 |
|  |  | (*b*) Page 1 |
| Introduction | | |
| Background/rationale | 2 | Page 1 |
| Objectives | 3 | Page 1,2 |
| Methods | | |
| Study design | 4 | Page 2,3 |
| Setting | 5 | Page 2 |
| Participants | 6 | (*a*) Page 3,Table 2 |
|  |  | (*b*) |
| Variables | 7 | Page 2,Table 1 |
| Data sources/ measurement | 8* | Page 1 |
| Bias | 9 | Page 6 |
| Study size | 10 | Page 6 |
| Quantitative variables | 11 |  |
| Statistical methods | 12 | (*a*) Page 3 |
|  |  | (*b*) |
|  |  | (*c*) |
|  |  | (*d*) |
|  |  | (*e*) |
| Results | | |
| Participants | 13* | (a) Page 1, Figure 1 |
|  |  | (b) |
|  |  | (c) |
| Descriptive data | 14* | (a) Page 4 |
|  |  | (b) |
| Outcome data | 15* | Page 4,Figure 4 |
| Main results | 16 | (*a*) Figure 2 /Table 4 |
|  |  | (*b*) |
|  |  | (*c*) |

| Other analyses | 17 | Page 4 |
| --- | --- | --- |
| Discussion | | |
| Key results | 18 | Page 3,4 |
| Limitations | 19 | Page 6 |
| Interpretation | 20 | Page 6 |
| Generalisability | 21 | Page 6,7 |
| Other information | | |
| Funding | 22 | Title Page |

*Give information separately for cases and controls.

**Note:** An Explanation and Elaboration article discusses each checklist item and gives methodological background and published examples of transparent reporting. The STROBE checklist is best used in conjunction with this article (freely available on the Web sites of PLoS Medicine at http://www.plosmedicine.org/, Annals of Internal Medicine at http://www.annals.org/, and Epidemiology at http://www.epidem.com/). Information on the STROBE Initiative is available at http://www.strobe-statement.org.
